# Advancing 3D-Printed Microfluidics: Characterization of a Gas-Permeable, High-Resolution PDMS Resin for Stereolithography

**DOI:** 10.3390/mi12101266

**Published:** 2021-10-18

**Authors:** Elyse Fleck, Alec Sunshine, Emma DeNatale, Charlise Keck, Alexandra McCann, Joseph Potkay

**Affiliations:** 1 VA Ann Arbor Healthcare System, Ann Arbor, MI 48105, USA; asunny@umich.edu (A.S.); denatale@umich.edu (E.D.); charjk@umich.edu (C.K.); mccanna@umich.edu (A.M.); 2 ECLS Laboratory, Department of Surgery, University of Michigan, Ann Arbor, MI 48109, USA

**Keywords:** microfluidics, microfabrication, stereolithography, additive manufacturing, 3D printing, poly(dimethylsiloxane)

## Abstract

The rapid expansion of microfluidic applications in the last decade has been curtailed by slow, laborious microfabrication techniques. Recently, microfluidics has been explored with additive manufacturing (AM), as it has gained legitimacy for producing end-use products and 3D printers have improved resolution capabilities. While AM satisfies many shortcomings with current microfabrication techniques, there still lacks a suitable replacement for the most used material in microfluidic devices, poly(dimethylsiloxane) (PDMS). Formulation of a gas-permeable, high-resolution PDMS resin was developed using a methacrylate–PDMS copolymer and the novel combination of a photoabsorber, Sudan I, and photosensitizer, 2-Isopropylthioxanthone. Resin characterization and 3D printing were performed using a commercially available DLP–SLA system. A previously developed math model, mechanical testing, optical transmission, and gas-permeability testing were performed to validate the optimized resin formula. The resulting resin has Young’s modulus of 11.5 MPa, a 12% elongation at break, and optical transmission of >75% for wavelengths between 500 and 800 nm after polymerization, and is capable of creating channels as small as 60 µm in height and membranes as thin as 20 µm. The potential of AM is just being realized as a fabrication technique for microfluidics as developments in material science and 3D printing technologies continue to push the resolution capabilities of these systems.

## 1. Introduction

The field of microfluidics has seen rapid growth in the last two decades [1], with some of the earliest successful examples being electrophoresis and gas chromatography developing into more current applications such as lab-on-a-chip devices [1,2]. Microfluidic devices contain feature sizes in the range of 1 to 500 µm and are constructed using microfabrication techniques in materials such as silicon, glass, plastic, and poly(dimethylsiloxane) (PDMS) [1].

PDMS is of particular interest, as it has become the most commonly used material for research-based microfluidic devices due to its chemical inertness, high gas permeability, low polarity, low electrical conductivity, elasticity, optical clarity, and transparency in the ultraviolet and visible regions [3]. PDMS can exhibit a broad range of mechanical properties depending on the crosslinking density of the network structure, resulting in both hard and soft types of PDMS [4,5].

In nearly all cases, soft lithography, a method in which a mold, usually constructed from photoresist on a silicon wafer, is used to form the desired features and thus microfluidic devices in PDMS. Soft lithography is simple, enables precise control over micron-scale features, and allows the creation of virtually any two-dimensional design. However, the resulting device is limited to the size of the mold, the creation of multilayer devices is difficult, and soft lithography is primarily a manual process [6]. For these reasons, scaling up soft lithography to larger device volumes and larger device sizes remains a challenge [7]. Further, truly three-dimensional (3D) devices cannot easily be created using soft lithography.

Recently, microfabrication via additive manufacturing (AM) has been realized, with advancements in 3D printing resolutions and capabilities [6,8,9,10,11]. Stereolithography (SLA), multijet printing (MJP), and fused deposition modeling (FDM) are the most explored methods of 3D printing microfluidics, with SLA exhibiting higher resolution, tighter tolerances, and compatibility with thermoset polymers compared to MJP and FDM.

A fourth approach, two-photon photolithography (2PP), is relevant to microfluidics for its highly resolved printing capabilities in the nanometer regime. However, this technique is still confined to small-scale, small build volume, and low-throughput applications due to its high cost and slow printing speeds, relative to other commercial 3D printers [12,13]. Further, the photocurable resins being developed for 2PP are not directly relevant to the work presented in this paper, as these materials utilize two-photon absorption from tightly focused laser pulses, confining the polymerization region to the focal volume of the lasers resulting in high-resolution prints. This is unlike SLA in which the resolution of the part is dictated by layer-by-layer fabrication and Beer’s absorption law of the resin to resolve small features [14,15].

While state-of-the-art SLA 3D printers harnessing digital micromirror technology have resolutions down to 2 µm in the XY plane and 1 µm in the Z plane [16,17], there is still a gap in material development, limiting the successful fabrication of truly microfluidic features. This is especially true for PDMS, as there are currently no high-resolution (<100 um features) PDMS–SLA resins that are commercially available or presented in the literature. To the best of our knowledge, the smallest membranes and channels successfully printed with PDMS resins via SLA are >330 µm and >1000 µm, respectively [18,19].

In this paper, we present the formulation of a high-resolution, gas-permeable, photopolymerizable PDMS resin and its successful implementation with a commercial SLA 3D printer to fabricate truly micron-scale parts with channels as small as 60 µm tall and membranes as thin as 20 µm.

## 2. Materials and Methods

### 2.1. Resin Formulation

Resin components were weighed out separately on a Quintix 125D-1S Semi-Micro Balance (Sartorius Lab Instruments GmbH & Co. KG, Goettingen, Germany) according to the desired *w*/*w*% of material. Components were combined and mixed by hand, heated for 2 h at 70 °C on a VMS-C7 S1 hot plate (VWR International, Radnor, PA, USA), and then sonicated with a Q700 sonicator (Qsonica LLC, Newtown, CT, USA) to ensure uniform mixing and particle size reduction. Sudan I was purchased from Sigma-Aldrich (St. Louis, MO, USA). 2-Isopropylthioxanthone (ITX) ≥ 98.0% was purchased from VWR International (Radnor, PA, USA). [7–9% (Methacryloxypropyl)methylsiloxane]-dimethylsiloxane copolymer (RMS-083) was purchased from Gelest, Inc. (Morrisville, PA, USA). 2, 4, 6-Trimethyl benzoyl diphenylphosphine oxide (TPO-L) was purchased from PL Industries of Esstech, Inc. (Essington, PA, USA).

The high-resolution formula for the resin described in this manuscript consists of a photoreactive methacrylate–PDMS copolymer of 98.6 *w*/*w*% RMS-083 with 0.8 *w*/*w*% TPO-L added as photoinitiator, 0.4 *w*/*w*% ITX added as a photosensitizer, and 0.2 *w*/*w*% Sudan I added as a photoabsorber. The Sudan I and ITX concentrations were chosen to maximize resolution while remaining below the solubility limit of the polymer. Various *w*/*w*% combinations of Sudan I and ITX were tested with the RMS-083 to determine the maximum amount of material that could be added while maintaining a uniform, stable resin. The *w*/*w*% of TPO-L was chosen based on previous work carried out by Bhattacharjee et al. looking at the solubility and curing parameters of the photoinitiator in RMS-083 [18].

### 2.2. Resin Characterization

Resin characterization was performed by placing uncured resin on a glass slide and cured by exposing a small circle of light from the printer (MAX X27 UV, Asiga, Alexandria, Australia) at 15 mW/m^2^ at various time points. The excess, uncured resin was rinsed from the glass slide with ACS Grade ≥99.5% isopropyl alcohol (IPA) (LabChem, Zelienople, PA, USA) purchased from Fisher Scientific Company (Hampton, New Hampshire, USA). The thickness of the cured resin was measured by taking side view images of the cured spot with the AM413T Dino-Lite Digital Microscope using the DinoCapture 2.0 software (Dunwell Tech, Inc., Torrance, CA, USA) (camera resolution is ±3 µm). Resins were cured and measured three separate times with triplicate measurements taken for each thickness (*n* ≥ 9). Regressions were run in both GraphPad and Excel to cross-check the regression results and to determine the slope and x-intercepts of these curves (and corresponding errors). A working curve describing the relationship between cure energy and cure thickness was input to the material file for printing with the Asiga MAX X27 UV printer (Asiga, Alexandria, NSW 2015).

### 2.3. Printing

All resin characterization and builds were printed using the commercially available Asiga MAX X27 UV printer (Asiga, Alexandria, NSW 2015). This printer uses digital light processing (DLP) technology with a 385 nm light source (wavelength range of 370–400 nm), an X and Y pixel resolution of 27 µm, and a Z (vertical) resolution of 1 µm. Asiga Composer Software version 1.2.11 (Asiga, Alexandria, NSW 2015) was used as the interface for handling STL files and controlling print parameters. All 3D models were generated in SOLIDWORKS (Dassault Systems, Waltham, MA, USA) and exported to the STL file format. The printed test channel structure was designed with an array of channels with varying heights and membranes with varying thicknesses; see Figure S1 for dimensions. The microfluidic channel junction was designed with channels that are 240 µm tall, 40 pixels (1080 µm) wide, and 2.7 mm long.

Glass slides were silanized with 3-(trimethoxysilyl)propyl methacrylate (Sigma Aldrich, St. Louis, MO, USA) following the procedure as described by A. Urrios et al. [20], then attached to the build platform using a UV epoxy (Proto Glass, Proto Products, Ashland City, TN, USA) at the start of each print to ensure adhesion of the build to the build platform. Contact angles were taken to validate the proper coating of the slides with the silane via a custom goniometer described previously [21]. The build platform was calibrated with the glass slide attached, and then printing proceeded as normal.

### 2.4. Print Post-Processing

Successful builds were removed from the build platform and soaked in IPA (Fisher Scientific Co., Waltham, MA, USA) to remove most of the uncured resin. For the test channels described below, a vacuum was applied to the open end of the channels to suction out the residual uncured, liquid resin. For the microfluidic channel junction, Silastic 2415569 Laboratory Tubing, 0.062” ID x 0.125” OD, 50’ (Cole Parmer, Vernon Hills, IL, USA) was attached to the inlet and outlet ports using 3140 RTV Silicone Conformal Coating (purchased from Ellsworth Adhesives, Germantown, WI, USA), then IPA was flowed through the channel junction at a rate of 0.2 mL/min using a Masterflex 7523-80 Peristaltic Pump (Cole Parmer, Vernon Hills, IL, USA) to ensure channels were completely void before testing. Flow channel and membrane dimensions were measured on the Dino-Lite Digital Microscope.

### 2.5. Mathematical Model of Dose Curves

A mathematical model previously described by Gong et al. [22] was used to create exposure dose curves for the resin. Exposure dose curves can predict the total exposure dose throughout the printed part as well as channel and membrane dimensions.

### 2.6. Absorbance

Absorbance measurements of 3D printing resins were taken from 300 to 600 nm using a Varian Cary 50 Bio UV-Vis spectrophotometer (Aligent Technologies, Santa Clara, CA, USA). Triplicates of each sample were run in Hellma^®^ absorption cuvettes (Hellma GmbH & Co., Müllheim, Germany) made of Herasil quartz with a spectral range of 260–2500 nm, pathlength of 10 mm, and chamber volume 3500 μL purchased from Sigma Aldrich (St. Louis, MO, USA). Absorbance data were normalized to a range of (0, 1).

### 2.7. Mechanical Testing

Mechanical testing was performed via tensile testing using a TA.XT*Plus* Texture Analyser and Exponent Connect software version 6 (Texture Technologies, Hamilton, MA, USA) at the Van Vlack Laboratory at the University of Michigan. Tensile bars were made according to ASTM D412 but scaled to fit the build area of the Asiga printer. A dimensioned drawing of the scaled tensile bars can be found in Figure S2. Resin samples were fabricated by printing tensile bars directly onto the build platform, removing the printed part, and washing in IPA before post-curing in an Asiga Flash-type DR-301C UV exposure chamber (Asiga, Alexandria, NSW 2015). Sylgard 184 (purchased from Ellsworth Adhesives, Germantown, WI) samples were formed by filling an acrylic mold (Figure S2) with 10:1 polymer to crosslinker mixture and baked for 1 h at 80 °C. Acrylic mold was formed by cutting out tensile bars from a 3.175 mm thick acrylic sheet (Professional Plastics, Inc., Fullerton, CA) using the Zing 16 laser engraver (Epilog Laser, Golden, CO, USA) and CorelDRAW 2017 software version 19 (Corel Corporation, Ottawa, ON, Canada). All tests were performed with at least *n* = 5. One-way ANOVA with a significance level of 0.05 and post hoc Tukey tests were performed using an online statistics calculator [23].

### 2.8. Gas Permeability

Gas permeability was tested using a custom, 3D-printed fixture (Figure S3), which permitted the application of a fixed pressure to one side of a thin film membrane and the measurement of oxygen concentration via a Milwaukee MW600 PRO Dissolved Oxygen Meter (Milwaukee Instruments Inc., Rocky Mount, NC, USA) in a fixed volume of DI water on the other side of the membrane. The custom fixture was drawn in SOLIDWORKS and printed using Asiga PlasGREY resin (Proto Products, Ashland, TN, USA) on the Asiga MAX X27 UV. PDMS resin-based films were printed on the Asiga MAX X27 UV printer. Sylgard 184 films were formed using an SCS G3P-12 Spin Coater (Specialty Coating Systems Inc., Indianapolis, IN, USA) with a 10:1 polymer to crosslinker mixture and baked for 1 h at 80 °C. All films were 100 µm thick and assembled into the membrane holder with the dissolved oxygen meter probe submerged in DI water. Oxygen was fed to the film with a constant pressure of 2 psi, and dissolved oxygen was measured for 90 min.

### 2.9. Percent Transmission

Transmission measurements (*n* = 3) were taken from 300 to 800 nm using a Varian Cary 50 Bio UV-Vis spectrophotometer (Aligent Technologies, Santa Clara, CA, USA). 100 µm films of the 3D printing resins were printed on the Asiga MAX X27 UV printer on uncoated glass slides to reduce surface roughness and ensure film transparency. IPA-soaked samples were soaked overnight for 18 h. Sylgard 184 films were formed using an SCS G3P-12 Spin Coater (Specialty Coating Systems Inc., Indianapolis, IN, USA), with a 10:1 polymer to crosslinker mixture and baked for 1 h at 80 °C. Results were normalized to the spectrum of the instrument’s light source.

## 3. Results

### 3.1. Resin Characterization

When formulating resins for SLA, it is critical that the photoresponsive components are compatible with (1) the light source of the printer and (2) the photopolymerizable polymer. The light source of the printer, Asiga MAX X27 UV, relies on a 385 nm LED restricting the selection of photoinitiators and photoabsorbers to spectra in this range. Further, the photopolymerizable PDMS selected for this custom resin is methacrylate based and proceeds most efficiently via a type I photopolymerization scheme. Building off of previous work carried out by Bhattacharjee et al. [18] and Gong et al. [22], we formulated a custom resin using RMS-083 as the photopolymerizable PDMS, TPO-L as the photoinitiator, Sudan I as the photoabsorber, and ITX as the photosensitizer. The resolution of this resin was enhanced by the novel combination of photoabsorber with a photosensitizer, imparting an electron transfer reaction to improve curing efficiency [15,24]. The best performing resin that we present in this paper in terms of resolution and stable composition is 0.2 *w*/*w*% Sudan I, 0.4 *w*/*w*% ITX, 0.8 *w*/*w*% TPO-L, 98.6 *w*/*w*% RMS-083, and is referred to as Sudan I + ITX. Other iterations of resin composition studied in this paper are given in Table S1 and chemical structures in Figure S4.

**Figure 1:**
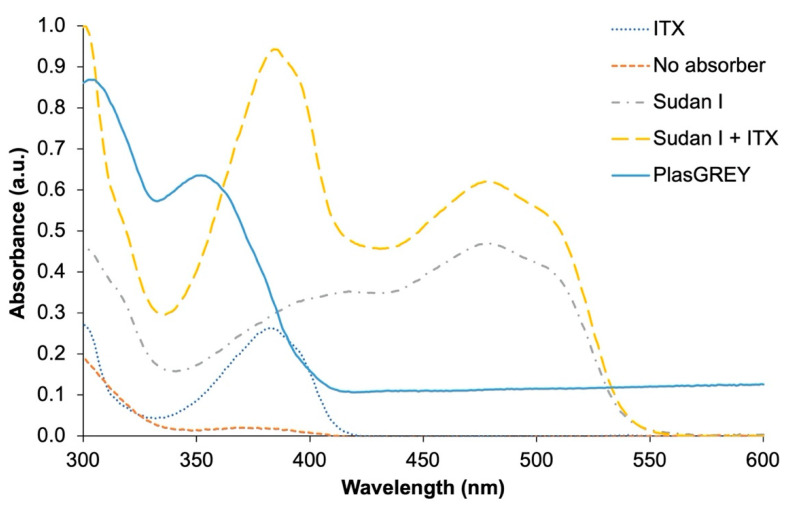
Absorbance spectra of various resin formulations compared with the commercially available PlasGREY resin. All custom resins have the same 0.8 *w*/*w*% of TPO-L and are made with RMS-083 polymer. Compositions of each formula can be found in Table S1.

To validate the compatibility of this resin formulation, Sudan I + ITX, with the Asiga MAX X27 UV printer, absorbance measurements of the resin were taken and are plotted in Figure 1. As shown, the custom resins containing both Sudan I and ITX absorb within the 385 nm range of the LED light source on the printer.

**Figure 2:**
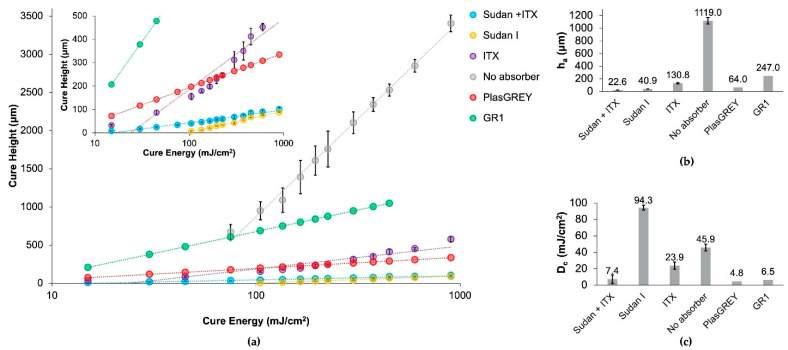
(**a**) Plots of cure height versus cure energy for custom resins with and without the UV absorbers, Sudan I and ITX, tested against commercial resins, GR1 and PlasGREY. The inset plot shows a zoomed-in view of the higher-resolution resins. Resin characterization data were collected at 385 nm on the Asiga MAX X UV27. Resin formulations Sudan I, ITX, Sudan I + ITX, and no absorber are all formulated with the same PDMS oligomers and same *w*/*w*% concentration of photoinitiator for comparison. Error bars represent the standard error of mean with a minimum of *n* ≥ 9 measurements per data point. GR1 and PlasGREY are high-resolution resins sold for use with the Asiga MAX 385 nm series. Data for these commercial resins were plotted from the corresponding material files provided upon purchasing the material, and therefore, no error bars are shown; (**b**) penetration depth values, *h_a_*, taken from the slope of each curve; (**c**) critical dose values, *D_c_*, calculated from the x-intercept of the working curve. Error bars represent standard error values from the regression fits (all fits had R^2^ ≥ 0.84).

A working curve using the spot testing method of cure energy vs. cure height was created (Figure 2a) to determine the penetration depth of the resin (1), where *α* is the absorbance of the material.

(1) $$h_{a}=\frac{1}{\alpha }$$

This curve was also used to develop the material file required for printing. The penetration depth, *h_a_*, describes how far light can penetrate into the resin during polymerization and can be determined by the slope of the working curve. Keeping optical dose constant, for a smaller *h_a_* (larger absorbance), the polymerization depth decreases, improving cure resolution. This analysis illustrates the relationship between the absorbance of the material and penetration depth, ultimately describing the resolution performance of the resin [22]. The characteristic penetration depths of various resin formulations can be found in Figure 2b, where Sudan I + ITX has the smallest *h_a_* value, validating the high-resolution of this custom formula.

In addition to the penetration depth, *h_a_*, the critical dose required for polymerization to form a non-flowable material, *D_c_*, or in other words, the amount of light required for polymerization to proceed until a solid material is formed [22], can be determined by calculating the x-intercept of the working curve in Figure 2a. The corresponding dose required for various resin formulations is given in Figure 2c. It is worth noting that possible sources of error in the resin characterization and resulting *h_a_* and *D_c_* values could be attributed to variation from batch to batch of the resin, the goodness of fit of the curves, and the microscope used for measuring cure height, which has a resolution limitation of 3 µm, making it difficult to measure thicknesses less than 10 µm.

As described by Gong et al. [22], the relationship between *D_c_* and *h_a_* is as follows:

(2) $$T_{c}=\frac{D_{c}}{I_{0}},$$

(3) $$z_{p}=h_{a}ln\frac{t_{p}}{T_{c}},$$

where in Equation (2), *I*_0_ is the instantaneous light intensity exposed by the printer. The dose received by the material, *D_c_*, is a function of light intensity and time, *T_c_*, where *T_c_* is the amount of time required for a non-flowable resin to form given the optical dose of light. In Equation (3), *z_p_* is the polymerization depth, and *t_p_* is the amount of time that light is being exposed to the material. Given the linear relationship between *h_a_* and *z_p_*, if a material has a smaller *h_a_* (or larger absorbance), the resin will be able to achieve higher resolution prints. However, it should be noted that polymerization depth, *z_p_*, does not equate to the printing resolution of the material. The *h_a_*, or penetration depth, is a better indication of resolution as it predicts the amount of light that can bleed into preceding layers (Z plane) and neighboring features in the XY plane [22].

A further indicator of resin performance is the efficiency of polymerization. This can be quantified by the critical dose, *D_c_*, where a smaller dose required to form a non-flowable resin has higher efficiency. As shown in Figure 2c, the combination of Sudan I + ITX results in a more efficient polymerization reaction than either Sudan I alone or ITX alone, which could lead to advantages such as shorter print times.

### 3.2. Theoretical Modeling

**Figure 3:**
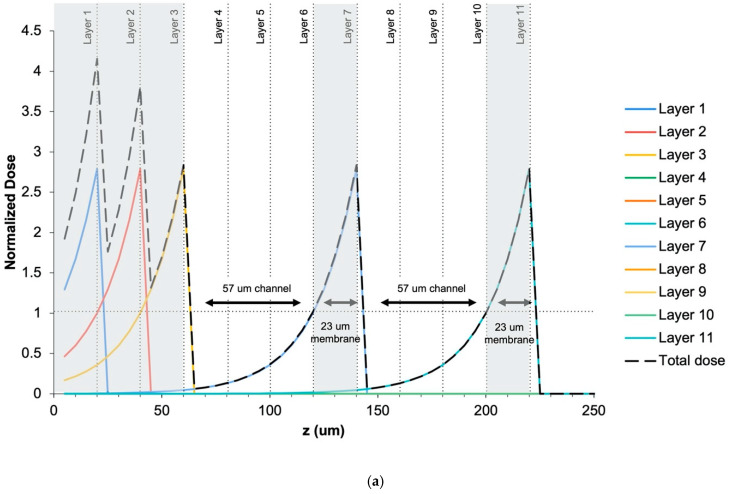

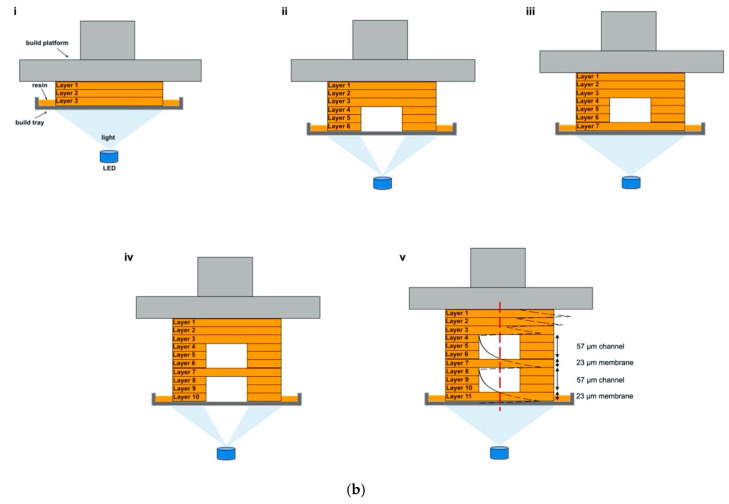
(**a**) Dose curve simulating 57 µm channels (designed with 60 µm) with 23 µm membranes (designed with 20 µm). Exposed layers are shaded grey; (**b**) schematic demonstrating corresponding curing pattern to the dose curve simulation where (i) corresponds to the first 3 layers of the pattern from (**a**), (ii) is the next three layers forming the first channel, (iii) layer 7 is the 23 µm membrane closing off the first channel, (iv) is the next three layers forming the second channel, (v) layer 11 closes off the second channel with a 23 µm membrane. The total normalized dose curve from (**a**) is overlaid on (v) after rotating 90 degrees. The vertical dashed line (red) indicates the region over which the dose curve was calculated. The Schematic is not to scale.

Before printing, theoretical work was carried out to validate the resolution performance of this resin using a previously developed mathematical model by Gong et al. [22]. This model describes the dose of light that individual layers of the part receive during a print, furthering our understanding of the polymerization of this custom resin. The data from the working curve in Figure 2a were used in this model to generate the dose curves shown in Figure 3a.

**Figure 4:**
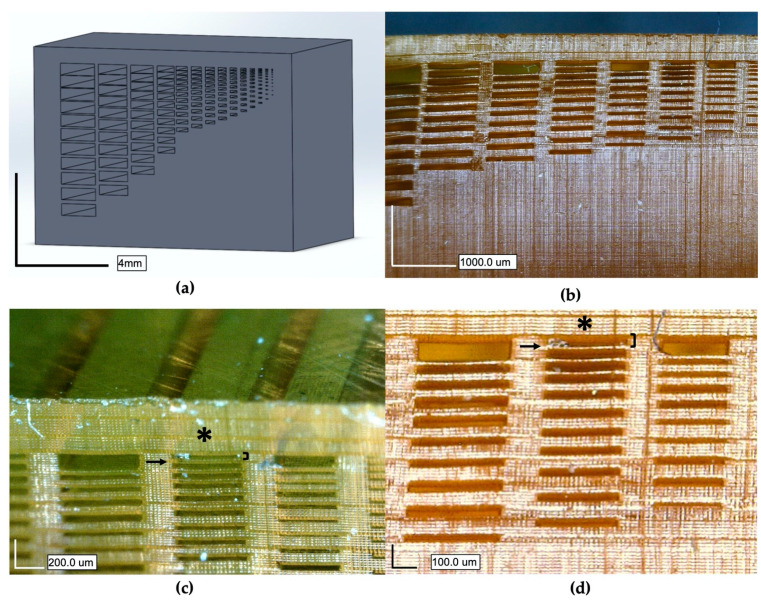
(**a**) A 3D CAD drawing of a test structure with microchannels of varying heights (10–300 µm) and membrane thicknesses (10–100 µm) to characterize resolution; (**b**) front view of the 3D-printed structure; (**c**) isometric, front view of the channels showing channels void of uncured resin along the top of the part; (**d**) close-up, front view of channels. Asterisk (*) denotes smallest printed channels successfully cleared of uncured polymer exposing 58.4 ± 1.7µm channel heights (denoted by brackets), with membranes as thin as 20.9 ± 0.5 µm (denoted by arrow).

This model was carried out applying the print parameters used for the printed test structure (Figure 4), and therefore, 20 µm print layers with 20 µm membranes and 60 µm channels were simulated in the plot. Given these parameters, the dose curves predict that a 57 µm channel with a 23 µm membrane can be successfully printed so that the total dose of the channel does not exceed 1—leaving it underexposed so that the unpolymerized material can be later flushed and cleared from the void. As described previously by Gong et al. [22], a dose of 1 is the amount of light required to polymerize a solid, or non-flowable material. In the case in the corresponding schematic in Figure 3b, layers 1, 2, and 3 are solid layers that receive a dose greater than 1 (Figure 3(bi)), layers 4, 5, and 6 have an unexposed section, leaving behind a 57 µm channel (Figure 3(bii)). Layer 7 received a dose greater than 1, forming a 23 µm layer or membrane (Figure 3(biii)) followed by an unexposed channel formed by layers 8, 9, and 10 (Figure 3(biv)). Layer 11 is exposed with a dose > 1, forming the final layer of the part (Figure 3(bv)).

### 3.3. Printing

To demonstrate the performance and resolution of this custom resin for microfluidic applications, a test channel structure was printed to determine the smallest channel and membrane that could be successfully fabricated and cleared of resin. Test channel heights ranged from 10 to 300 µm tall and from 90 to 2700 µm wide, and membranes ranged from 10 to 100 µm thick—a dimensioned schematic is given in Figure S1. As shown in Figure 4, and in concurrence with the math model in Figure 3, our custom resin is capable of printing and clearing channels as small as 60 µm tall, 540 µm wide, and 6.54 mm long, and membranes as thin as 20 µm (drawn/expected dimensions). See Supplementary Table S2 for more data comparing drawn versus actual channel and membrane dimensions. This is a notable increase in z-resolution over previously reported work with PDMS–SLA resins, in which the highest resolved channel heights were 1.2 mm tall with 330 µm membranes [18].

**Figure 5:**
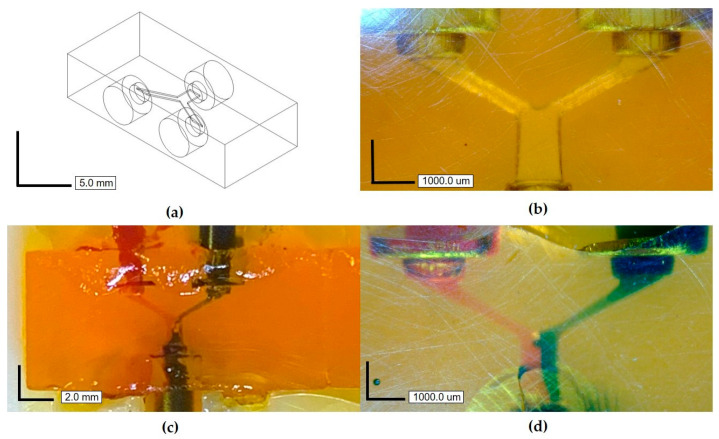
(**a**) A 3D CAD drawing of a microfluidic channel junction with 240 µm tall and 40 pixels wide (1080 µm) channels; (**b**) zoomed-in top view of the cleared channels in the 3D-printed structure; (**c**) top view with red- and blue-dyed DI water entering the channel junction and exiting as a purple fluid; (**d**) zoomed-in top view image showing red- and blue-dyed DI water mixing at the exit port.

To further demonstrate the relevance and printability of this custom resin, a basic microfluidic channel junction with 240 µm tall, 40-pixel wide (1080 µm), and 2.7 mm long channels was printed with Sudan I + ITX resin, as presented in Figure 5. A solution of DI water with red and blue food dye was run through the channels to visualize flow in the channels.

### 3.4. Mechanical Testing

**Figure 6:**
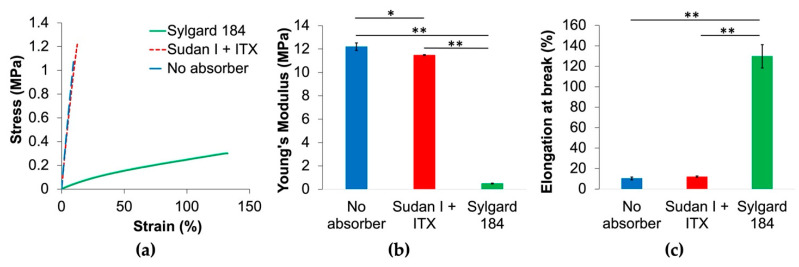
(**a**) Stress–strain curve from tensile testing of Sylgard 184 (green), Sudan I + ITX resin (red), and no absorber resin (blue); (**b**) Young’s modulus of the same three materials; (**c**) elongation at break for each material. In (**b**,**c**), a one-way ANOVA test was performed to determine statistical significance. A single asterisk (*) signifies *p* < 0.05, and a double asterisk (**) signifies *p* < 0.01 for post hoc Tukey’s test between groups. Error bars represent the standard error of mean (*n* = 5).

Mechanical testing was performed to compare the properties of Sudan I + ITX resin, no absorber resin, and Sylgard 184. The stress–strain curves, elongation at break, and Young’s modulus are given in Figure 6.

### 3.5. Gas Permeability and Optical Clarity

**Figure 7:**
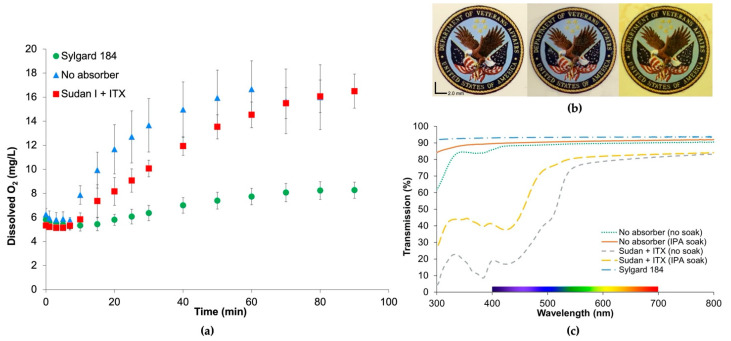
(**a**) Plot of dissolved oxygen in DI water through a 100 µm thick film, demonstrating the permeability of the 3D-printed resin relative to Sylgard 184. Error bars represent the standard error of mean (*n* = 3); (**b**) images of 100 µm Sylgard 184 film (**left**), no absorber film (**middle**), and Sudan + ITX film (**right**) laid over an image with text and color demonstrating optical clarity of resin; (**c**) transmission spectrum of 100 µm film of Sylgard 184, no absorber films before and after soaking in isopropyl alcohol (IPA) to remove extractables, and Sudan + ITX films before and after soaking in IPA.

The oxygen permeability of the custom cured resin was explored by applying a fixed pressure to one side of a 100 µm thick membrane and measuring dissolved oxygen over time in a fixed reservoir of DI water on the other side of the membrane. The relative oxygen permeability of Sudan I + ITX, no absorber, and Sylgard 184 is revealed in Figure 7a by the dissolved oxygen curves as a function of time. In general, a larger permeability will result in a faster rise in dissolved oxygen and a larger final dissolved oxygen value. Given the nearly twofold increase in dissolved oxygen of the 3D-printed films relative to Sylgard 184, SEM images were taken from the no absorber and Sudan + ITX films to validate the films did not have pinholes and that no fracture or deformation to the films occurred during the 3D printing process or testing. As seen in Figure S5, the films have no evidence of damage from 3D printing or the testing setup or of pinholes, verifying that the oxygen measurements are a result of the permeability of the PDMS films.

The optical clarity of this resin is demonstrated in Figure 7b, in which 100 µm films of cured resin are overlayed across colored images with text. The % transmission of the same films from 7b is given in Figure 7c, validating the transparency of this resin in the colored region of the spectra. The transmission of the cured films in the range of 300 to 550 nm was enhanced after soaking in IPA overnight to remove extractables from the polymer.

## 4. Discussion

Given the widespread use of PDMS in the fabrication of microfluidics, creating a high-resolution photocurable PDMS resin was a significant motive in the development of this formulation. Further, the Asiga MAX X27 UV printer was a desirable system for microfabrication for its high-resolution and open material system.

To achieve the high-resolution printing performance required to print truly microfluidic devices, the unique combination of a photoabsorber, Sudan I, and photosensitizer, and ITX was implemented in this formula. We found that the combination of these two compounds improved resolution over the formulas with photoabsorber alone and photosensitizer alone. From Figure 2b, the Sudan + ITX resin has the smallest penetration depth, *h_a_* = 22.6 µm versus *h_a_* = 40.9 µm for Sudan I (no ITX) and *h_a_* = 130.8 µm for ITX (no Sudan I). The resolution capabilities of this Sudan I + ITX resin demonstrates a twofold increase over the absorber-only formula and a fivefold increase over the photosensitizer-only formula.

The curing efficiency of the resin was greatly enhanced by the combination of these two compounds, as shown by the *D_c_*, where Sudan + ITX requires the smallest dose, *D_c_* = 7.4 mW/cm^2^, for polymerization to form a non-flowable material. This greatly enhanced the curing efficiency over the Sudan-I-only formula and marginally improved curing efficiency compared with the ITX-only formula. Generally, it can be concluded that Sudan I is the main driver in resolution, and ITX dictates curing efficiency during polymerization.

The printability of this resin was first studied using a theoretical dose curve model to predict the optimal layer thickness for printing and corresponding channel heights given the penetration depth of Sudan I + ITX. An in-depth explanation of the math and theory of these dose curves can be found in a study by Gong et al., who developed this model [22]. The dose curves in Figure 3a were plotted to mimic and validate the printed test channel structure, as shown in Figure 4. As the plot verifies, a 20 µm layer is thick enough to prevent bleeding of light into the preceding layers so that a dose of 1 is never reached, forming a channel.

While the printer is capable of 1µm resolution in the z plane and 27 µm in the XY plane, the actual printing resolution is determined by the material and the system combined. From the results of the math model and the test channel print in Figure 4, we printed with a 20 µm layer thickness, producing channels that were resolved within 2 µm of the expected 60 µm height and membranes within 1µm of the expected 20 µm thickness (see Table S2). The minimum feature size in the XY plane for which the channel could be cleared of uncured resin was 540 µm for 60 µm tall channels. However, channels as small as 30 µm tall and 270 µm wide appear to be resolved in the XY plane (Figure 3b, third channel from the right), but we were unable to clear them of resin. We believe that had taller channels been printed with this width, then channels would have been properly formed and cleared.

The smallest printable channel height for this formula was 60 µm with 20 µm membranes, as shown in Figure 4. Potentially smaller channels and membranes could be printed given the high resolution of this resin. However, the large viscosity of the resin restricts the printability of smaller feature sizes due to high separation forces. For comparison, the viscosity of the commercially available GR1 resin is 700 cP whereas the Sudan + ITX resin presented in this paper has a much larger viscosity of 5960 cP (Figure S6). Separation forces on the printed part is a common mode of failure for SLA systems, where the viscous forces of the resin create a suction-cupping effect on the face of the part and will peel or tear layers away during the build [25]. Intuitively, the larger the viscosity of the resin is, the more challenging these separation forces will be to overcome.

Further, the high viscosity of this material makes it difficult to remove unpolymerized resin from the micron-scale channels. The capillary forces within the channel can be overcome with the use of solvents to dilute the resin and mechanical stimulation such as sonication or the application of pressurized air, but this puts the printed part at risk of failure while it is still in the semi-reacted “green” state—the state of the part before post-curing to polymerize any unreacted groups after printing [26,27]. Applying too much force when trying to remove the uncured resin can result in the separation of layers or fracture of the thin membranes. In the case of the microfluidic channel junction printed in Figure 5, larger channels (240 µm tall) were printed for ease of removing the uncured resin and to avoid damaging the part. The next steps in the development of this material will include the addition of a diluent to reduce the viscosity of the material, thereby improving the printability and ease of post-processing.

As demonstrated in Figure 6, the mechanical properties of this material are brittle compared with Sylgard 184, whereas Young’s modulus is much larger for both custom formulas, and elongation at break is much smaller. The inclusion of the photoabsorber and photosensitizer have little effect on the brittleness of the material, revealing that the bulk mechanical properties are driven by the polymer, RMS-083. For microfluidic applications where a low modulus is required, e.g., pneumatic pump, this material would not be suitable. However, in one regard, the larger Young’s modulus of this material aided in overcoming the large separation forces experienced during printing from the high viscosity.

In addition to the mechanical properties of this resin, oxygen permeability and optical clarity were investigated to validate this resin for other desirable features of PDMS. Figure 7a validates the permeability of this cured resin to oxygen given the increase in dissolved oxygen content in DI water over time. The Sudan I + ITX and no absorber films exhibited larger dissolved oxygen content compared with Sylgard 184, validating its functionality as a gas-permeable material.

In similarity to Sylgard 184 and the no-absorber films in Figure 7b, the Sudan + ITX resin maintains the visual acuity of the text, and the colors of the background image are still distinguishable despite the orangish-hue present from the photoabsorber Sudan I. In Figure 7c, transmission in the colored region of the spectrum reaches 83% at 695 nm and allows >75% transmission in the 500–800 nm range. The lower transmission in the UV range of the spectrum can be attributed to the photoabsorbing compounds still present in the polymer and could be addressed by soaking in IPA for a longer time or using another solvent to remove additional extractables, further improving transparency.

## 5. Conclusions

We successfully demonstrated the formulation of a gas-permeable and high-resolution, photopolymerizable PDMS resin with the novel combination of photosensitizer and photoabsorber to print *truly* micron-scale features (sub 100 µm). Further, the compatibility of this resin with a commercial system illustrates that automation and the high throughput of microfluidic devices are within reach. With more innovation and development on resins and photoresponsive material systems, microfabrication techniques will no longer be restricted by the laborious processes used today.

## Data Availability

Data will be provided via requests to the corresponding author.

## Supplementary Materials

The following are available online at https://www.mdpi.com/article/10.3390/mi12101266/s1, Figure S1: Test channel STL, Figure S2: Mechanical testing, Figure S3: Dissolved oxygen meter membrane holder assembly, Table S1: Resin compositions and colors, Figure S4: Resin composition, Table S2: Printing resolution, Figure S5: SEM images of permeability-tested films, Figure S6: Rheology.
