# Supplementary material for: Advancing 3D-Printed Microfluidics: Characterization of a Gas-Permeable, High-Resolution PDMS Resin for Stereolithography
Source: Micromachines (Basel). 2021 Oct 18;12(10):1266. doi: 10.3390/mi12101266 (PMC8540252; doi:10.3390/mi12101266)

Supplementary Materials

Figure S1. Test channel STL.

Dimensioned drawing of test channel part. Channel heights range from 10 to 300 µm tall and from 90 to 2700 µm wide, and membranes range from 10 to 100 µm thick. All dimensions in the drawing are in mm.

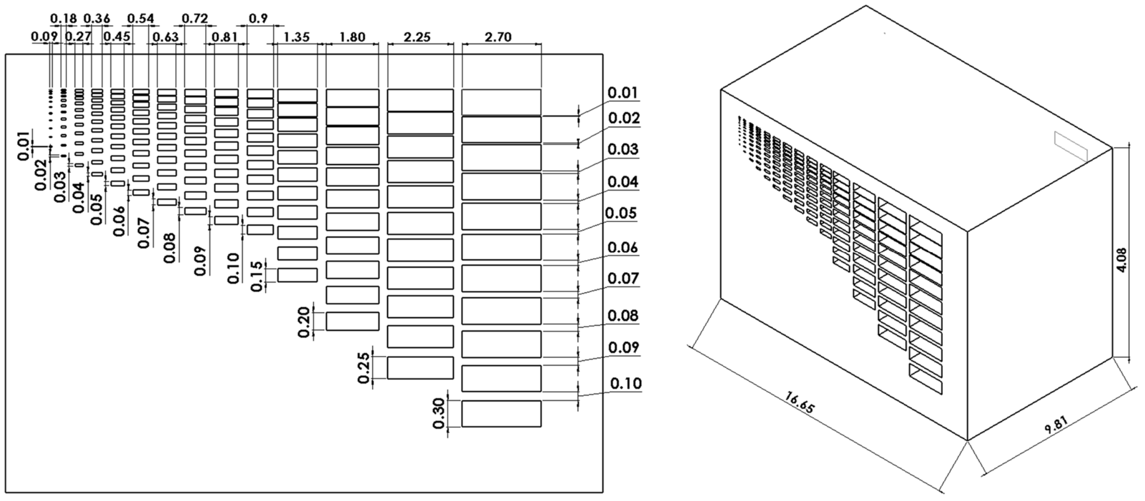

Figure S2. Mechanical testing.

Dimensioned drawing of tensile bars printed for mechanical testing (left). Bars were drawn according to ASTM D412 die C and a 0.44x scalar was applied so that the bars would fit within the build area of the Asiga MX X27 UV printer. The acrylic mold used to fabricate Sylgard 184 samples is shown on the right.

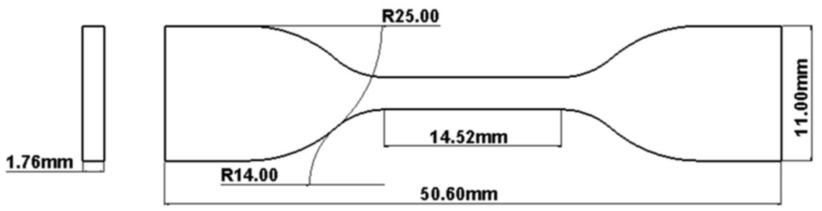

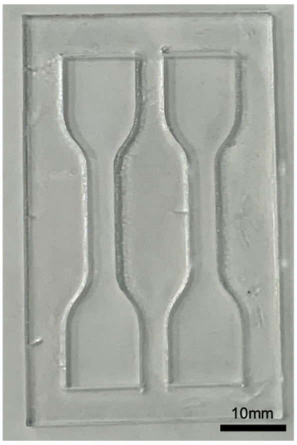

Figure S3. Dissolved oxygen meter membrane holder assembly.

CAD drawings for the 3D-printed membrane holder (top and bottom left) and actual assembly of the membrane holder and dissolved oxygen meter (top and bottom right).

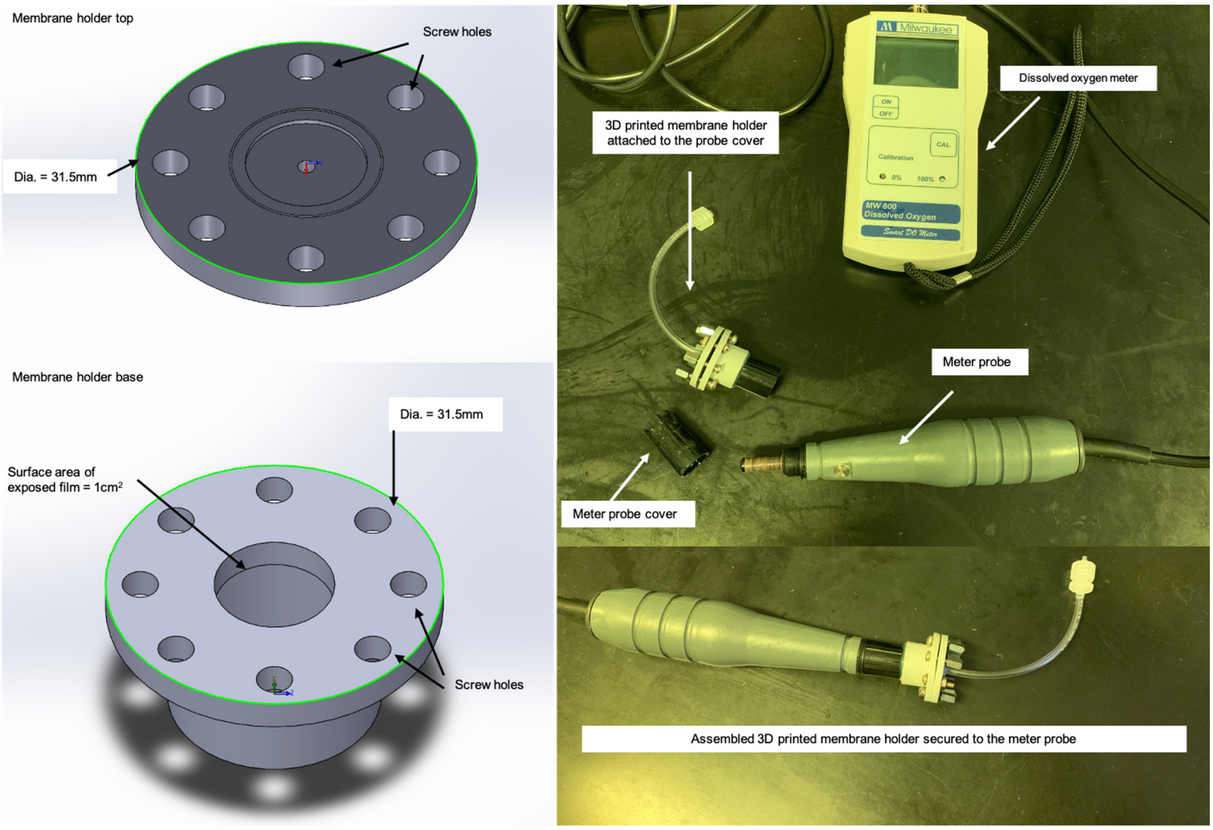

**Table S1.** Resin compositions and colors.		
**Resin**	**Formula**	**Color**
GR1	Commercial resin produced by Pro3dure	Colorless
PlasGrey	Commercial resin produced by Asiga	Grey
No absorber	99.2 w/w% RMS-083, 0.8 w/w% TPO-L	Colorless
Sudan I	99.0 w/w% RMS-083, 0.8 w/w% TPO-L, 0.2 w/w% Sudan I	Orange
ITX	98.8 w/w% RMS-083, 0.8 w/w% TPO-L, 0.4 w/w% ITX	Colorless
Sudan I + ITX	98.6 w/w% RMS-083, 0.8 w/w% TPO-L, 0.2 w/w% Sudan I, 0.4 w/w% ITX	Orange

Figure S4. Resin composition.

Chemical structures of the components used in the formulas presented in this paper and w/w% are given in Table S1. [7-9% (Methacryloxypropyl)methylsiloxane] - dimethylsiloxane copolymer (RMS-083) (top left), 2, 4, 6-Trimethyl benzoyl diphenylphosphine oxide (TPO-L) (top right), 2-Isopropylthioxanthone (ITX) (bottom left), Sudan I (bottom right).

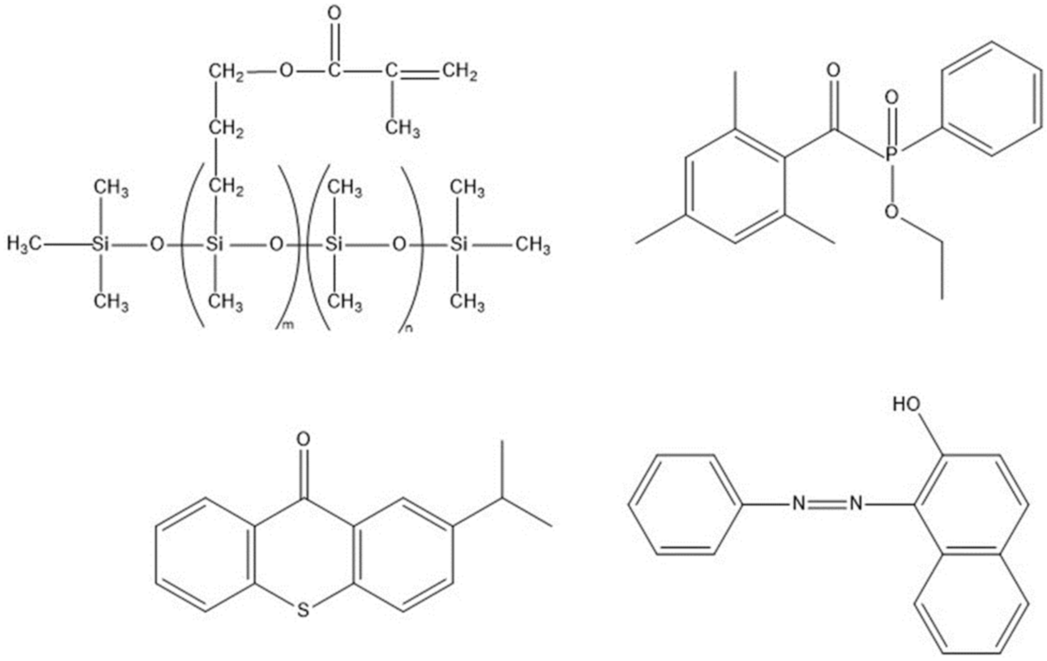

Table S2. Printing Resolution. Expected vs actual dimensions of the channels in the printed structure in Figure 4. Note n ≥ 3 for all measurements. Error is the standard error of mean.

	Expected	Actual
Channel Height	60	58.43 ± 1.72
	150	146.13 ± 1.73
	200	208.82 ± 4.94
Membranes	20	20.94 ± 0.54
	40	42.80 ± 2.31
	60	61.11 ±1.51
	80	78.19 ± 3.23
	100	99.88 ± 1.77
Channel Width	270 (10 pixels)*	270.20 ± 9.10
	540 (20 pixels)	500.00 ± 4.37
	810 (30 pixels)	729.8 ± 10.10

* Did not clear of resin.

Figure S5. SEM images of permeability-tested films.

Images were taken on a Tescan Rise field emission SEM (Tescan, Czech Republic) at the Michigan Center for Materials Characterization (University of Michigan, Ann Arbor, MI). Samples were gold coated for 40 seconds using an SPI Module Carbon Sputter Coater (West Chester, PA, USA). Secondary electron images were taken in high vacuum, at 5kV. Beam's current was 100 pA. During imaging, energy-dispersive X-ray spectroscopy (EDS) was performed on the EDAX Octane Elite (Ametek, Berwyn, PA) to identify unknown deposits and debris on the film surface.

SEM images increase in magnification from left to right. Neither film showed any signs of fracture or deformation that would result in a significant change in permeability. The artifacts visible on the no absorber film are debris such as dust and hair. In the middle and right images, the dendritic deposits are from the electrolyte solution used to calibrate the dissolved oxygen meter and were identified using the EDS on the SEM. The Sudan + ITX film also shows some debris such as dust and hair. There are slight striations across the film, likely from scratches on the build platform on to which these films were printed.

No absorber:

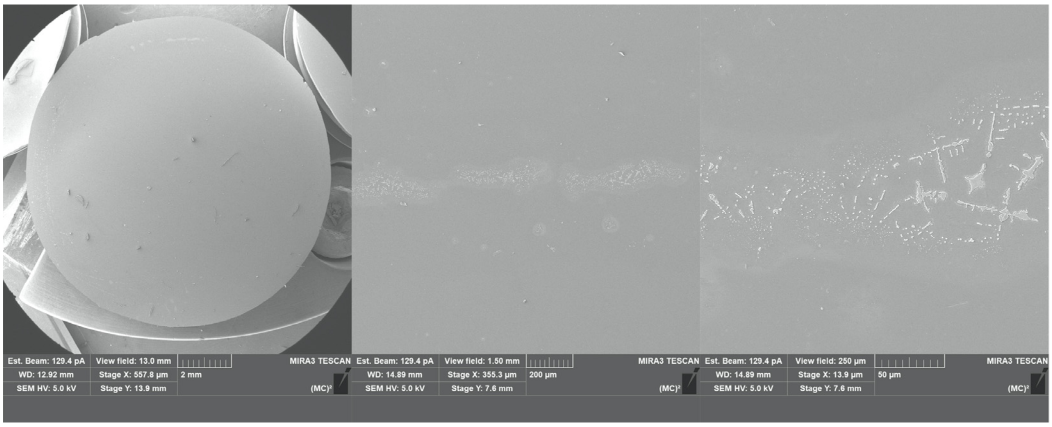

Sudan + ITX:

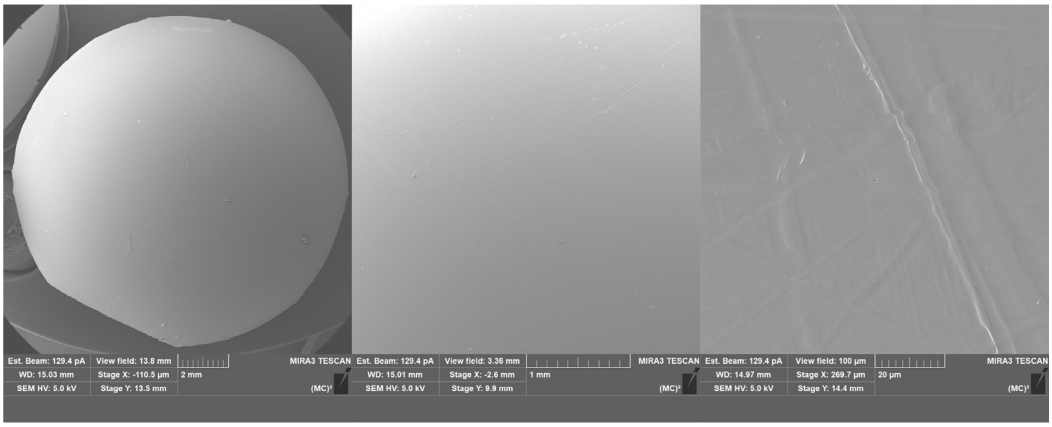

Figure S6. Rheology.

The viscosity of resins was determined using a TA Discovery HR-2 Rheometer (TA Instruments, New Castle, DE) at the University of Michigan's Battery Lab, Ann Arbor, MI. A flow sweep of the samples was run using a 20mm parallel Peltier steel plate and a 1mm gap.

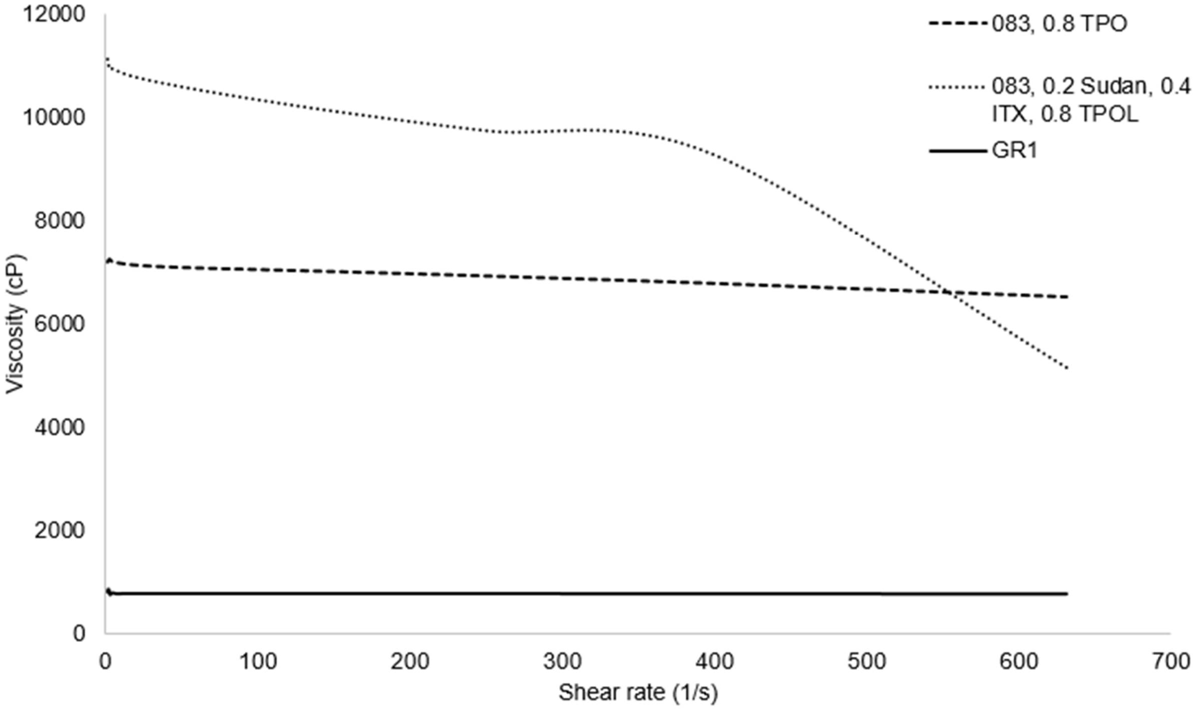

Supplement: Supplementary file 1 [file micromachines-12-01266-s001.zip › micromachines-1373642-supplementary.pdf]
